# Effect of a BSHP process on clinical work ability of nurses in a children's cardiac intensive care unit

**DOI:** 10.3389/fped.2023.1143855

**Published:** 2023-05-26

**Authors:** Fangyan Ma, Aiguo Shi, Lanzheng Bian, Mei Li, Mingxiong Li, Banghong Xu

**Affiliations:** Cardiothoracic Surgery, Children's Hospital of Nanjing Medical University, Nanjing, China

**Keywords:** children, cardiac intensive care unit, nursing, nursing handover, communication, quasi-experimental study

## Abstract

**Background:**

Nurse shift change is the key step to ensuring the integrity, dynamics, and continuity of clinical nursing in intensive care units.

**Objectives:**

To evaluate the effect of a bedside shift handover process (BSHP) on the clinical work ability of first-line clinical nurses in a children's cardiac intensive care unit (CICU).

**Methods:**

This quasi-experimental study was performed on the first-line clinical nurses working in a pediatric CICU at Children's Hospital of Nanjing Medical University between July and December, 2018. Participants were trained by the BSHP. This article is based on the STROBE checklist.

**Results:**

A total of 41 nurses were trained, with 34 women. The nurses in the intensive care unit showed significantly improved clinical work ability, including the ability to assess illness/find problems, master professional knowledge, standardized hands-on ability, ability to express communication, strain handling capacity, and humanistic care and accomplishment (all *P* < 0.05), after training.

**Conclusion:**

BSHP might improve the clinical work ability for pediatric CICU nurses through a standardization shift handover. The traditional oral shift change in CICU can easily cause an information distortion, and it is difficult or even impossible to arouse the nurses' enthusiasm. This study suggested that BSHP might be an alternative shift change process for pediatric CICU nurses.

## Introduction

Nurse shift change refers to a process in which one group of nurses communicates the current or potential symptoms and information on all aspects of treatment and care of the children under their care to the group of nurses taking over (1). This process is the key step to ensuring the integrity, dynamics, and continuity of clinical nursing in intensive care units (ICUs) (2–4). It provides a very important platform for nurses to discuss nursing issues interactively, gain team support, exchange patients' conditions, and judge whether nursing methods need to be changed, and can also improve the critical thinking of nurses (5).

Children in the cardiac ICU (CICU) are seriously ill. The traditional oral shift change can easily cause an information distortion (6, 7). Communication stagnation or obstacles are easy to occur in the shift change process, and these obstacles will also lead to adverse nursing events and clinical risks (8).

How to mobilize the subjective initiative of nurses has become a hot topic in nursing management. Medical errors kill tens of thousands of patients each year and represent US$42 billion of medical expenditure annually worldwide (9). In a 2004 report by the United States Joint Review Committee on Accreditation of Healthcare Organizations (JCAHO), it was pointed out that 72% of the causes of infant injury or death were related to communication barriers among medical staff and failure to communicate effectively (10). Errors caused by communication barriers in hospitals have a serious impact on the safety and quality of life of children (11, 12). Therefore, it is significant to improve the communication mode of nurse shift change and reduce the adverse events for children caused by poor communication among nurses.

Therefore, early intervention and treatment are carried out to avoid detrimental events (13). At the same time, it can also improve the active thinking ability of junior nurses thereby that the quality of shift handover has a specific and controllable inspection standard, hence both sides of shift handover can quickly grasp the illness of children and ensure the overall and specific content of shift handover (14).

According to the research of the JCAHO, more than 60% of clinical adverse events are caused by communication barriers (15, 16). In traditional shift handover, the nurse's focus completely falls on the part of nursing that is closely related to the nurse. Therefore, the overall understanding of the condition of the children in custody, the mastery of clinical signs, and the current priority work are neglected (17). On the other hand, implementing a standardized, evidence-based communication model (SBAR)-based communication mode for children's CICU can encourage nurses to hand over the shift contents of critically ill children to the successor nurses clearly and accurately. By using this method, not only the nurses can handover the shift and carry out the nursing work according to the item sequence of the form, but also they can have organized thoughts in the nursing work for children and make their levels clear, fully understanding the important parts of the shift. In addition, the nurse who takes over can also collect information according to the form, analyze and summarize the situation of the children, and master and raise questions at different levels, promoting effective communication. The JCAHO recognizes the efficiency of standardized communication (18). Therefore, it is necessary to formulate an appropriate bedside shift mode for CICU children (19, 20). The SBAR was proposed by the World Health Organization (21). SBAR refers to Situation (S), Background (B), Assessment (A), and Recommendation (R). SBAR can effectively and quickly transfer information and avoid omission (22, 23). The subjective initiative of nurses can be fully mobilized to ensure the smooth progress of nursing work, thus will improve the efficiency of teamwork (24).

## Research question

Hence, this study aimed to evaluate the effect of a bedside shift handover process (BSHP) based on the SBAR communication tool on the clinical work ability of first-line clinical nurses in a children's cardiac intensive care unit (CICU).

## Materials and methods

### Study design and participants

This quasi-experimental study was performed on the first-line clinical nurses working in a pediatric CICU at Children's Hospital of Nanjing Medical University between July and December, 2018.This experimental study was conducted mainly among nurses working in the front line of the clinic. The main idea is to study the clinical work competencies of nurses working in ICU. These competencies include ability to assess illness/find problems;mastering professional knowledge;standardized hands-on ability;ability to express communication;strain handling capacity;humanistic care and accomplishment. The work competencies of the nurses were determined by examining these aspects of the nurses.These contents cover all aspects of clinical nurses' working ability, and the assessment is more comprehensive. It is more able to assess the actual working ability of nurses, and also to find out the deficiencies in clinical work and the abilities that need further improvement.Inclusion criteria:1. Nurse professional certificate; 2. Nurses working in ICU; 3. Nurses willing to cooperate with clinical research; Exclusion criteria: 1. Sick leave, maternity leave; 2. Nurses who do not work in ICU; 3. Nurses with mental health problems; 4. Unwilling to participate in the study.

### Intervention

#### Design of the BSHP

Based on the SBAR communication mode, a shift handover table (from now on, referred to as the “shift table”) was designed (Supplementary Table S1). The shift table includes four sections. S represents the basic situation of the child at present, such as bed number, name, weight, and age, also vital signs (T, p, HR, BP, and SPO_2_). B represents the background, e.g., diagnosis and operation, the specific date, the breathing mode of the child (spontaneous or mechanical ventilation), and vital signs. In addition, it also includes the positive symptoms and signs, the critical degree of the disease, the abnormal examination indexes, the treatment currently given, and the nursing measures already taken. A represents the current situation of the children's input and output, such as the situation of each drainage tube. Finally, R represents the current special situation of children, such as what problems have occurred, the measures needed to solve these problems, and the issues that need the attention of the next nursing staff.

#### Training with the shift table

Between July 1 and 10, 2018, the department set up a special team, with the head nurse of the ICU of the children's cardiothoracic surgery department as the core. During the training process, the ability of nurses should be developed from the following aspects, including: the ability to observe and evaluate illness/find problems; master professional knowledge; standardized hands-on ability; expression and communication can remain (patients, doctors, nurses); strain handling capacity; humanistic care and accomplishment/shop, guidance (group). The training was conducted through the unified study of all clinically responsible nurses, the analysis of common cases in this department, and the on-the-spot shift exercise and situational situation mode conducted by the CICU bedside for children (20).

The teaching and training of all CICU nurses were carried out several times and repeatedly for common clinical methods. This training mainly included explaining the definition of this communication mode, the clinical advantages of this communication mode, the significance of this communication, and how to use the shift table to carry out the shift handover. The head nurse and the monitoring team leader gave timely guidance and urged improvement of the existing problems and weaknesses. SBAR video materials were played to consolidate the theoretical knowledge of the nursing staff. Simulated SBAR scenarios of typical clinical cases were organized to deepen the learning impression in the form of sitcoms. Therefore, understanding and mastery of this communication mode by nurses could be improved. Finally, the nurses were evaluated on their understanding and using the shift table (25, 26).

#### Implementation of the shift table

The position of each station is very important during nursing handover. The nurse handing a patient over should stand on the left side of the patient. The nurse taking charge of the patient should stand on the right side of the child patient (i.e., beside the bedside table). At the end of the bed, the head nurse of the ICU or the leading head nurse and other nurses are standing. The handover scenario includes S: name, medical record number, age, and vital signs (T, p, HR, BP, and SPO_2_); B: primary diagnosis, chief complaint, current breathing pattern, positive symptoms and signs, critical degree of illness, abnormal test and examination indexes, current treatment, and adopted nursing measures; A: basic conditions and special conditions of the child on duty in this round, the abnormal results, the mental state, breathing and circulation conditions, whether there is a wet or cold sensation on the skin (Because when we touch the skin temperature of extremities of children with congenital heart disease (such as fingers or toes), if the touch sensation of the skin of the child is wet and cold but not warm at this time, it indicates that the extremity endings of the children are not perfused enough, and further explains the poor cardiac function), what kinds of tubes the child currently has, how to fix and observe them, whether a ventilator is used, timely assessment of extubation evidence, input and output, and changes in the disease; R: summarize the relevant recommendations of the nurse for the handover to the child. In addition, according to the specific condition of the child, the nurse suitable for succession is proposed, the treatment and corresponding nursing measures that the nurse should need, and the special succession under special circumstances, e.g., whether intubation is needed, whether catheterization is needed, whether the child suffers from pneumothorax, etc. At the same time, the nurses who need to take over must pay serious attention to the issues (27, 28). During shift handover, the bedside handover was conducted according to the shift handover scenario, and the conditions of the children, tubes, and skin were carefully checked. The responder must complete the work and ensure that all items are on standby. When there is a complex condition or the transfer of items is unclear, must be immediately clarify the problems, and the cause must be immediately identified and dealt with in a timely manner. Otherwise, the successors have the right not to transfer. After the shift, the head nurse of the ICU or the leading head nurse commented on the shift's problems, weaknesses, and high-risk links.

Two quality control groups of ICU management quality and patient care quality were created to conduct an irregular inspection (The head nurse randomly selected two patients a day from Monday to Friday to examine the patient's transition process), analysis, feedback, and rectification. In addition, the quality was continuously improved, and timely feedback was given to all nurses during the department's learning period, hence further adjustments and feedback could be made the following month.

### Outcomes

The primary outcome was the grip of the clinical first-line responsible nurse on the patient's condition can be evaluated. The evaluation standard was the clinical work ability assessment record form revised by the nursing department of our hospital in January 2016 (Supplementary Table S2). It included nurses' and children's data, a cross-section of assessment practice, and an assessment of clinical abilities with scoring (1) ability to assess illness/find problems, (2) master professional knowledge, (3) standardized hands-on ability, (4) ability to express communication, (5) strain handling capacity, and (6) humanistic care and accomplishment, along the score of each variable was 3, 2, 2, 1, 1, and 1 point respectively, with a total score of 10 points, whereas increments of 0.2 points for assessment. In this study, the nurses' comprehensive clinical work competency was used as the independent variable, while the handover form developed after the SBAR model, this was part of the dependent variable. The change of the developed handover form and the handover pattern were used to promote the change of the nurses' comprehensive clinical work competency.

The secondary outcomes were effective communication, efficient communication, mastering the disease condition of children, responsibilities clarified, and team cooperation. The nurse of the children's CICU must fully understand all the conditions of the children taken over in one day, including disease condition, treatment plan, pipeline, management of input and output, etc. Using the clinical fact-finding table of the nurse's condition in the hospital (Supplementary Table S2), the head nurse of the ICU and the leading head nurse of the same day asked about the nurse's condition of the children during the bedside quality control inspection. Before (January 2018) and six months after (January 2019) the implementation of the shift table, the nurses in the ICU were investigated and compared on their ability to assess the condition of children and find problems, master professional knowledge points, standardize their hands-on ability, express and communicate ability, hard-board handling ability, humanistic care quality, etc. According to the Chinese version of the nurse shift change evaluation scale, which is based on the Chinese version of the English version NASR (Nursing Assessment of shift Report) scale developed by Lin et al. (29) and Sand-Jecklin et al. (30), the responsible nurses were investigated for shift change effect evaluation before and after the implementation of the shift table.

### Statistical method

SPSS 21.0 (IBM, Armonk, NY, USA) was used to carry out the statistical analysis of the data. Continuous data conforming to the normal distribution (according to the Shapiro-Wilk test) were expressed as means ± standard deviation. The effect evaluation after implementing the shifty table was compared using the paired t-test. Categorical variables were presented as *n* (%). Two-sided *P*-value < 0.05 was considered statistically significant.

### Ethics

This work has been carried out in accordance with the Declaration of Helsinki (2000) of the World Medical Association. The study protocol was approved by the ethics committee of Children's Hospital of Nanjing Medical University. Written informed consent was obtained from all participants.

## Results

**Figure 1:**
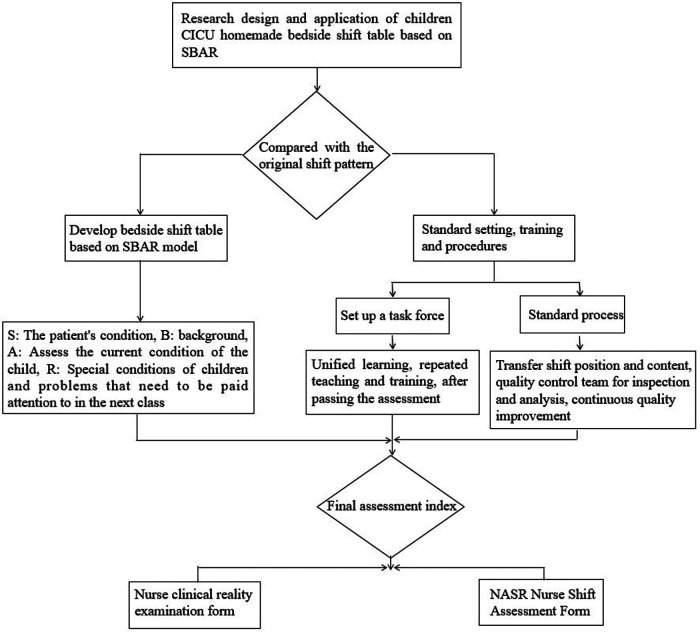
Workflow. Figure is the flow chart of the research design, and the application of the Chinese table is based on the SBAR pattern for the transfer of children. In the figure, we can see that the transfer table is designed after comparing it with the traditional transition mode. Then they began to set standards and train responsible nurses. After the training, the final assessment will be carried out.

All responsible nurses were trained for one month and the assessment began the second month after the training. The workflow of this study was shown in Figure 1. Among the 41 nurses who participated in the training, there were 34 women and 7 men. Among them, 11 (26.8%) were in-charge nurses, 18 (43.9%) were senior nurses, and 12 (29.3%) were nurses. There was one graduate (2.4%), 28 undergraduates (68.3%), and 12 with a junior college degree (29.3%) nurses. There were 16 nurses with <5 working years (39.0%), 19 with 5–10 working years (46.3%), and six with >10 working years (14.6%).

After training, 41 copies of the assessment scale were issued, 41 were recovered, and 41 were valid questionnaires, for an effective rate of 100%. In primary outcome, the nurses in the ICU showed significantly improved clinical workability after being trained by BSHP, including the ability to assess illness/find problems (2.7 ± 0.2 vs. 2.4 ± 0.2), master professional knowledge (1.4 ± 0.6 vs. 1.0 ± 0.4), standardized hands-on ability (1.5 ± 0.1 vs. 1.1 ± 0.6), ability to express communication (0.5 ± 0.1 vs. 0.4 ± 0.1), strain handling capacity (0.5 ± 0.1 vs. 0.4 ± 0.1), and humanistic care and accomplishment (0.5 ± 0.2 vs. 0.4 ± 0.3) (all *P* < 0.05) (Table 1).

**Table 1:** Primary outcome.

Clinical workability	Before training (*n* = 41)	After training (*n* = 41)
Ability to assess illness/find problems	2.4 ± 0.2	2.7 ± 0.2^*^
Mastering professional knowledge	1.0 ± 0.4	1.4 ± 0.6^*^
Standardized hands-on ability	1.1 ± 0.6	1.5 ± 0.1^*^
Ability to express communication	0.4 ± 0.1	0.5 ± 0.1^*^
Strain handling capacity	0.4 ± 0.1	0.5 ± 0.1^*^
Humanistic care and accomplishment	0.4 ± 0.3	0.5 ± 0.2^*^

* *P* < 0.05.

Furthermore in secondary outcome, effective communication (4.7 ± 0.6 vs. 3.8 ± 0.5), efficient communication (4.7 ± 0.5 vs. 3.8 ± 0.5), mastering the disease condition of children (4.7 ± 0.5 vs. 4.0 ± 0.7), responsibilities clarified (4.6 ± 0.6 vs. 4.1 ± 0.6, *P* < 0.05), and team cooperation (4.1 ± 0.4 vs. 3.6 ± 0.5) were all significantly improved (all *P* < 0.05) after the implementation of the BSHP (Table 2).

**Table 2:** Secondary outcomes.

Secondary outcomes	Before training (*n* = 41)	After training (*n* = 41)
Promoting effective communication	3.8 ± 0.5	4.7 ± 0.6^*^
Efficient communication	3.8 ± 0.5	4.7 ± 0.5^*^
Mastering the disease condition of children	4.0 ± 0.7	4.7 ± 0.5^*^
Responsibilities clarified	4.1 ± 0.6	4.6 ± 0.6^*^
Team cooperation	3.6 ± 0.5	4.1 ± 0.4^*^

* *P* < 0.05.

## Discussion

This study suggested that BSHP might improve clinical workability, effective communication, efficient communication, mastering the disease condition of children, responsibilities clarified, and team cooperation for pediatric CICU nurses through a standardization shift handover.

Effective communication between health care professionals is inevitable and important factor in clinical decision making (31).The reason is that nurses, physicians and other healthcare professionals constantly face situations and conditions that needs properly and on time communication. Ineffective communication leads to unsatisfactory outcomes for patients (17). States that one of the five factors that inhibit effective communication between health care professionals is the multiple transfers of patients, together with the complexity of today's health care system.These factors confuse the healthcare personnel (32). Unfortunately, these complexities exist in all healthcare settings and so several solutions are needed to overcome them. One of these solutions is to identify and meet educational needs of healthcare personnel.

Nurses are required to be trained about effective communication skills. The importance of proper training of SBAR technique show that they have reduced unwanted hospital accidents and losing patients' information by using this technique (21). Also, studies reported that proper use of communication methods decrease the errors in the site of surgery; improve the transfer process; and increase patient safety. As the results of this study show, BSHP, based on the SBAR communication mode, significantly increase the communication skills of nurses. It means that BSHP should be used in the teaching of communication skills for nurses.

However, as emphasized by Firiesen et al., organizational support and commitment of mangers are two essential factors in successful usage of SABR technique. This support should contain providing learning opportunities, technical and official supports, supervision, and feedback.In this culture, the nurses should encourage to talk about the actual or potential problems they have in communication with themselves and with other healthcare staff. As we know, any problems in effective communication among health care professionals may create many problems and put patients at risk of harm. Therefore, this culture helps to protect patient safety and prevent unwanted hospital accidents.

After the authors' department implemented the improved SBAR-based standardized bedside shift handover table, unified and standardized management was carried out on the content of the shift table, which further standardized the shift handover process. At the same time, all the nurses in the CICU received clarifications on the specific positions and their respective clinical duties during shift handover. The responsible nurses of the shift handover carefully handed over the contents of the handover and its related key points item by item according to the template's contents. The patient's condition must be clear, organized, and focused, including the eventual problems such as circulation, input, output, and relevant suggestions. The successors are responsible for carefully and comprehensively checking the condition of the children they are becoming responsible for, thus improving the critical thinking of nurses, the work has subjective initiative, and can take the initiative to pay attention to the situation of patients. Therefore, the omission of key and high-risk links (such as the positive signs of the patient, the condition of peripheral circulation, the effectiveness of drainage tube, etc.) for critically ill children is reduced, and the situation of inattention and failure to grasp key points during shift change is reduced. Therefore, the nurse showed improved scores on the general situation, main disease condition, diagnosis, treatment, key points of specialized nursing, and overall nursing quality of the children compared with before the implementation of the shift table. This novel process is conducive to the correct transmission of information about the disease condition, treatment, nursing, and other aspects of the children during the shift handover. The nurses not only have to master the current vital signs of the children and the treatment and medication care but also have to have an understanding of the abnormal values of relevant laboratory tests and blood gas analysis and of the changes in the patient's condition, which would help the responsible nurses to discover the changes in the patient's condition earlier. The implementation of the shift table is conducive to the first-line responsible nurses to fully understand the early identification of disease change indicators in the children. By reminding each other of the current or potential safety hazards of the children during shift change, the head nurse and the nursing team leader check each other at every level so that the quality of shift change and the good cooperation ability of the whole nursing team is improved.

Implementing the SBAR-based communication model for children's CICU to make the shift table not only standardizes the bedside handover process for critically ill children with congenital heart diseases but also ensures the comprehensiveness and accuracy of children's information during the handover process. At the same time, it also promoted the cooperation of nursing teams during the shift. Based on the SBAR communication mode, the children's CICU shift table mobilized the subjective initiative of front-line nurses to think positively. Nonetheless, it also improves their analytical and critical thinking abilities on the condition, treatment, and nursing of the children under their care. More importantly, it can promote effective communication between medical care and nursing, and ensure the safety of children and the safety of overall medical care.

## Limitations

This study has limitations. The sample size was small since the study was performed in a single department of a single hospital. The generalizability and exportability of the shift table are unknown. Even though the nursing quality was evaluated using validated tools, the impact of the implementation of the shift table on hard metrics (e.g., morbidity, complications, mortality, etc.) was not evaluated. The time taken for each patient's handover was not evaluated. Due to the COVID-19 epidemic situation, no trial or analysis of clinical responsibility nurses has been carried out, and further research will be considered at a later stage.

## Conclusions

In conclusion, BSHP might improve clinical workability, effective communication, efficient communication, mastering the disease condition of children, responsibilities clarified, and team cooperation for pediatric CICU nurses through a standardization shift handover, which may reduce the threat to patient safety during nursing. Therefore, BSHP might have the value of promotion.

## Data Availability

The original contributions presented in the study are included in the article/**Supplementary Material**, further inquiries can be directed to the corresponding author/s.
