# Supplementary material for: Effect of a BSHP process on clinical work ability of nurses in a children's cardiac intensive care unit
Source: Front Pediatr. 2023 May 26;11:1143855. doi: 10.3389/fped.2023.1143855 (PMC10250624; doi:10.3389/fped.2023.1143855)
Supplement: Supplementary file 1 [file Table1.docx]

**Supplementary Table S1.** Bedside shift schedule in cardiothoracic surgery intensive care unit

Bed No Name Name of operation Body weight kg Age

| Date | Days before/after operation | Ventilator CPAP nasal oxygen inhalation | Special medication | General run of things | Drainage | Special case |
| --- | --- | --- | --- | --- | --- | --- |
|  |  | Exposed cannula: cm |  | HR: Times /min | Chest tube output: ml |  |
|  |  |  |  | BP: mmHg |  |  |
|  |  |  |  | CVP: cmH_2_O | Gastric juice: ml |  |
|  |  |  |  | Peripheral: | Urine: ml |  |
|  |  |  |  | In: ml  Out: ml | Stool: ml |  |
|  |  | Exposed cannula: cm |  | HR: Times /min | Chest tube output: ml |  |
|  |  |  |  | BP: mmHg |  |  |
|  |  |  |  | CVP: cmH_2_O | Gastric juice: ml |  |
|  |  |  |  | Peripheral: | Urine: ml |  |
|  |  |  |  | In: ml  Out: ml | Stool: ml |  |
|  |  | Exposed cannula: cm |  | HR: Times/min | Chest tube output: ml |  |
|  |  |  |  | BP: mmHg |  |  |
|  |  |  |  | CVP: cmH_2_O | Gastric juice: ml |  |
|  |  |  |  | Peripheral: | Urine: ml |  |
|  |  |  |  | In: ml; Out: ml | Stool: ml |  |
|  |  | Exposed cannula: cm |  | HR: Times /min | Chest tube output: ml |  |
|  |  |  |  | BP: mmHg |  |  |
|  |  |  |  | CVP: cmH_2_O | Gastric juice: ml |  |
|  |  |  |  | Peripheral: | Urine: ml |  |
|  |  |  |  | In: ml  Out: ml | Stool: ml |  |
|  |  | Exposed cannula: cm |  | HR: Times /min | Chest tube output: ml |  |
|  |  |  |  | BP: mmHg |  |  |
|  |  |  |  | CVP: cmH_2_O | Gastric juice: ml |  |
|  |  |  |  | Peripheral: | Urine: ml |  |
|  |  |  |  | In: ml  Out: ml | Stool: ml |  |
|  |  | Exposed cannula: cm |  | HR: Times /min | Chest tube output: ml |  |
|  |  |  |  | BP: mmHg |  |  |
|  |  |  |  | CVP: cmH_2_O | Gastric juice: ml |  |
|  |  |  |  | Peripheral: | Urine: ml |  |
|  |  |  |  | In: ml  Out: ml | Stool: ml |  |

**Supplementary Table S2.** Record form for examination of nurses’ clinical work ability

Hospitals: Department (Detailed Name)

Examination time: Year Month day Time Signature of examiner:

| 1. Nurse information | | | | | | | | | | | | | | | | | | | | | | | | | |
| --- | --- | --- | --- | --- | --- | --- | --- | --- | --- | --- | --- | --- | --- | --- | --- | --- | --- | --- | --- | --- | --- | --- | --- | --- | --- |
| Name |  | | Age | | |  | | | Highest degree | |  | | | | | | | The length of professional life | | | | | |  | |
| Years of service | ≤5 years | | 6-10 years | | | ≥11years | | | Post | | Nurse 口 Team leader 口 Head nurse 口 | | | | | | | | | | | | | | |
|  | | | | | | | | | | | | | | | | | | | | | | | | | |
| Gender |  | | Age | | |  | | | Diagnosis | | | | |  | | | | | Level of care | | | |  | | |
| 2. cross-section of assessment practice (Mark:”√“, single choice or multiple choice is allowed) | | | | | | | | | | | | | | | | | | | | | | | | | |
| Shift change | | Infusion | | | | | | Gastrointestinal decompression | | | | | | | Nasal feeding/atomization | | | | | | | Measure vital signs or blood sugar | | | |
| Preoperative/Post-operative Care | | Turn over/skin care | | | | | | Catheter/wound care | | | | | | | Oral care | | | | | | | Record Intake and output | | | |
| Rescue a patient | | ECG monitoring | | | | | | Instrument usage | | | | | | | Assist in getting out of bed/handling | | | | | | | Pre-screening triage | | | |
| Health education | | Health guidance | | | | | | Transfer to/from hospital | | | | | | | Other | | | | | | | | | | |
| 3. Assessment of nurses’ clinical ability | | | | | | | | | | | | | | | | | | | | | | | | | |
| Major competency items (gradient 0.2 points) | | | | | | | | | | | | Fraction | | | | | Score | | | Comments on Existing Problems | | | | | |
| The ability to observe and evaluate illness/find problems | | | | | | | | | | | | 3 | | | | |  | | | Questions of principle:  Subsection questions: | | | | | |
| Master professional knowledge | | | | | | | | | | | | 2 | | | | |  | | |  |  |  |  |  |  |
| Standardized hands-on ability | | | | | | | | | | | | 2 | | | | |  | | |  |  |  |  |  |  |
| Expression and communication can remain (patients, doctors, nurses) | | | | | | | | | | | | 1 | | | | |  | | |  |  |  |  |  |  |
| Strain handling capacity | | | | | | | | | | | | 1 | | | | |  | | |  |  |  |  |  |  |
| Humanistic Care and Accomplishment/Shop, Guidance (Group) | | | | | | | | | | | | 1 | | | | |  | | |  |  |  |  |  |  |
| Total score | | | | | | | | | | | |  | | | | |  | | |  |  |  |  |  |  |
| 4 .Evaluation of Results (Fill in Values) | | | | | | | | | | | | | | | | | | | | | | | | | |
| Unqualified (<6) | | | |  | | Qualified (6-6.9) | | | |  | | | Good (-8.9) | | | | | | |  | Excellent (9-10) | | | |  |
| Attached: difficulty coefficient (mark”√“and average value) | | | | | | | | | | | | | | | | | | | | | | | | | |
| Degree of Difficulty | | | | | 0.9 | | 0.95 | | 1 | | | | | | | 1.05 | | | | | | 1.1 | | | |
| Cross-section | | | | |  | |  | |  | | | | | | |  | | | | | |  | | | |
| Patient condition | | | | |  | |  | | Primary care or moderate dependence | | | | | | | Severe, after major surgery, etc | | | | | | Critical illness+complications | | | |
| Mean value of difficulty coefficient | | | | |  | |  | | Real score (one decimal place) | | | | | | | | | | | | |  | | | |
| Team professional ability | | | | | Excellent | | | | Good | | | | | | | Medium | | | | | | Bad | | | |
| Suggestions for further training and assessment improvement | | | | |  | | | | | | | | | | | | | | | | | | | | |
